# Electrospun Zr-Doped CaO Sorbent for CO_2_ Capture

**DOI:** 10.3390/nano13040747

**Published:** 2023-02-16

**Authors:** Vyacheslav V. Rodaev, Svetlana S. Razlivalova, Alexander I. Tyurin, Vladimir M. Vasyukov

**Affiliations:** Institute for Nanotechnology and Nanomaterials, Derzhavin Tambov State University, Internatsionalnaya Str. 33, 392000 Tambov, Russia

**Keywords:** CaO nanofibers, electrospinning, cyclic carbonation/decarbonation, CO_2_ uptake capacity, microstructure, phase composition, mechanical properties

## Abstract

A Zr-doped CaO sorbent for high-temperature CO_2_ capture was fabricated using electrospinning. The nanofiber sorbent with an average filament diameter of about 160 nm is characterized by an initial CO_2_ uptake capacity of 12.1 mmol/g, a specific surface area of 79 m^2^/g, an indentation Young’s modulus of 520 MPa, and a hardness of 1.6 MPa. After 50 carbonation/decarbonation cycles, the sorbent has a decent CO_2_ uptake capacity of 9.7 mmol/g due to the uniform distribution of CaZrO_3_ in the CaO nanofibers to prevent CaO grain growth caused by CaCO_3_ sintering. It is revealed that the sorbent CO_2_ uptake capacity decreases both with an increase in the decarbonation temperature and with an increase in the CO_2_ concentration in the gas flow upon carbonation, where the sorbent CO_2_ uptake capacity is more sensitive to the decarbonation temperature than to the CO_2_ concentration in the gaseous stream during carbonation. It is assumed that the electrospun regenerable Zr-doped CaO sorbent is effective for removing CO_2_ from flue gases.

## 1. Introduction

Fossil fuel combustion for steel production, power generation, and transportation is the main reason for carbon dioxide accumulation in the atmosphere. CO_2_ is the main greenhouse gas causing global warming, which is accompanied by serious climate issues. According to the Mauna Loa Observatory (Waimea, HI, USA), the atmospheric CO_2_ concentration reached an all-time high of 421 ppm in 2022. For the foreseeable future, the global economy will require an even greater consumption of fossil fuels, which will negatively impact the environment due to a further increase in the atmospheric CO_2_ concentration. Therefore, the objective of reducing CO_2_ emissions into the atmosphere has become very important. Technology for carbon capture and sequestration (CCS) is one of the most promising solutions for removing CO_2_ from exhaust gases [1]. CCS technology consists of three steps: the capture, transportation, and storage of CO_2_, where CO_2_ capture is the most costly step [2].

Amine scrubbing based on CO_2_ absorption is the most commonly used CO_2_ capture technology on the industrial scale [3]. However, this technology has significant disadvantages: low operating temperature requiring the pre-cooling of hot exhaust gases, high regeneration energy, absorbent degradation, and the corrosive nature of amine solutions [3,4].

The reversible physical adsorption of CO_2_ using solid adsorbents and the pressure swing adsorption, thermal swing adsorption, or temperature vacuum swing approach is seen as a promising alternative to amine scrubbing [5,6]. Carbonaceous materials (activated carbon, carbon molecular sieves, carbon nanotubes, and graphene), zeolites, metal–organic frameworks, and silica materials are conventional adsorbents that are extensively studied for CO_2_ adsorption [5,6]. Usually, they show a high CO_2_ uptake capacity, but at temperatures below 100 °C and significant pressure.

Recently, the calcium looping (CaL) process based on the reversible reaction between CaO and CO_2_ has attracted great attention as an alternative technology for large-scale CO_2_ capture [7]. The advantages of the CaL process over amine scrubbing are the lower cost of CO_2_ capture, a lower energy penalty, the wide range of operating temperatures of CaO, the fast sorption kinetics and high sorption capacity of CaO, the environmental safety of the spent materials, and the possibility of their further use, for example, in the cement industry [7].

The main disadvantage of the CaL process is that natural and inexpensive CaO significantly and rapidly loses its ability to sorb CO_2_ as the number of carbonation/decarbonation cycles increases due to the intense sintering of the resulting CaCO_3_ [4,8]. Thus, cost-competitive synthetic CaO-based sorbents resistant to multicyclic carbonation/decarbonation with a high CO_2_ uptake capacity are of interest. Among the most studied and quite effective approaches to enhancing the performance of CaO-based sorbents is incorporating CaO particles into inert supports with a high Tammann temperature [7,9,10]. There are many ways to fabricate such sorbents [7,9,10]: the sol–gel method, coprecipitation, flame spray pyrolysis, the impregnation method, mixing, etc.

During the carbonation reaction, a CaCO_3_ layer grows on the surface of CaO particles, making some of the CaO inside the particles inaccessible to CO_2_, since the diffusion coefficient for CaO is two orders of magnitude greater than for CaCO_3_ [11]. In [12], the critical carbonate layer thickness was estimated at about 50 nm. This fact indicates the expediency of obtaining nanostructured CaO-based sorbents.

It is well known that electrospinning, a versatile, simple, inexpensive approach and one of the most efficient nanofiber fabrication technologies, is rapidly developing in several directions to expand its capability of creating functional nanofiber materials for various applications: catalysis [13]; air and wastewater purification [14,15]; energy harvesting, conversion, and storage [16]; gas sensors [17]; tissue repair and regeneration [18]; etc. One is production on a large scale using the multi-nozzle method or the free surface technique [19]. Another is to create complicated approaches through multiple-fluid electrospinning processes, such as coaxial [20], triaxial [21], side-by-side [22], and other processes. The third is to combine it with other chemical and physical methods to further convert electrospun filaments to functional nanofibers [23,24]. The fabrication of CaO nanofibers via the heat treatment of electrospun composite filaments containing an oxide precursor and a polymer, carried out in our previous work, is a fine example of the third direction [25].

The aim of this work was to obtain, for the first time using electrospinning, a composite nanostructured CaO sorbent for high-temperature CO_2_ capture and to study the evolution of its microstructure, CO_2_ uptake capacity, and mechanical properties under cyclic carbonation/decarbonation conditions.

## 2. Materials and Methods

### 2.1. Materials

Polyacrylonitrile with a molar mass of 150,000 g/mol (PAN, Sigma-Aldrich, Saint Louis, MO, USA) as a binder polymer, calcium and zirconium acetylacetonates (CaAA, ZrAA, Sigma-Aldrich, Saint Louis, MO, USA) as precursors of calcium oxide and zirconium dioxide, and, finally, dimethylformamide as a solvent were used.

### 2.2. Preparation

The procedure for obtaining nanofibrous chemical adsorbents of carbon dioxide included three stages: (a) the preparation of a solution suitable for electrospinning; (b) the electrospinning process; (c) the heat treatment of electrospun composite fibers to obtain the final oxide nanofibers. To prepare a composite solution, CaAA and ZrAA were dissolved in a freshly prepared 10 wt.% PAN solution with stirring at 50 °C until a transparent solution was obtained. The concentrations of CaAA and ZrAA in the electrospun solution were varied within 4.1–4.0 wt.% and 0–3.5 wt.%, respectively, to obtain final nanofibers with a Zr/Ca molar ratio in the range of 0–0.3.

Electrospun composite fibers collected as non-woven mats were fabricated using a NANON-01A electrospinning machine (MECC, Fukuoka, Japan) at room temperature. The selected electrospinning parameters, such as a voltage of 14 kV between the injector tip and the collecting electrode, a distance between them of 12 cm, and a solution feed rate of 0.8 mL/h, made it possible to obtain cylindrical composite fibers with a smooth surface.

All fabricated mats of composite filaments were annealed in a muffle furnace at 800 °C for 1 h in an air atmosphere. The heating rate was 1 °C/min to prevent damage to the filaments during CaAA, ZrAA, and PAN decomposition.

### 2.3. Characterization

The thermal analyzer EXSTAR TG/DTA7200 (SII Nano Technology, Tokyo, Japan) was used in the thermogravimetric mode to measure the CO_2_ uptake capacity of the fabricated CaO-based nanofibers. As-prepared nanofibers were carbonated at 620 °C for 30 min in a gaseous stream containing 15 vol.% CO_2_ and 85 vol.% N_2_. The gas flow with 15 vol.% CO_2_ corresponds to the exhaust gases of coal-fired power plants [26]. After that, the filaments were decarbonated at 800 °C for 20 min under a N_2_ flow. Further, they were cooled to 620 °C in the gaseous stream containing 15 vol.% CO_2_ and 85 vol.% N_2_ for carbonation. The carbonation/decarbonation cycle was repeated 50 times. The carbonation and decarbonation temperatures were the same as in [25]. The CO_2_ uptake capacity of the nanofibrous sorbents was determined by the amount of captured CO_2_ divided by the sample weight before carbonation.

XRD patterns were recorded using a D2 Phaser X-ray diffractometer (XRD, Bruker AXS, Karlsruhe, Germany) and identified using the PDF-2 Diffraction Database File compiled by the International Centre for Diffraction. The microstructure of the nanofibers was analyzed using a JCM-7000 scanning electron microscope (SEM, Jeol, Tokyo, Japan). We also used SEM to calculate the average nanofiber diameter as well as the average grain size. XRD and SEM measurements were carried out at room temperature. Nitrogen adsorption isotherms were recorded using an Autosorb iQ-C gas sorption analyzer (Quantachrome Instruments, Boynton Beach, FL, USA) to calculate the specific surface area of nanofibrous sorbents using the Brunauer–Emmett–Teller method.

To examine the mechanical characteristics of nanofiber mats, a TI-950 nanotriboindenter (Bruker AXS, Karlsruhe, Germany) was used. Their indentation Young’s modulus and hardness were determined at room temperature using a spherical indenter made of zirconia ceramic. The radius of curvature was many times larger than the size of the macropores formed by randomly arranged filaments. The indentation Young’s modulus and the hardness of the nanofiber mats were calculated from the load–displacement curve according to the Oliver–Pharr method [27]. The tested samples of nanofiber mats were fixed with ethanol on the polished surface of zirconia ceramic pellets with a Young’s modulus and hardness many orders of magnitude higher than those of nanofiber mats.

## 3. Results and Discussion

**Figure 1:**
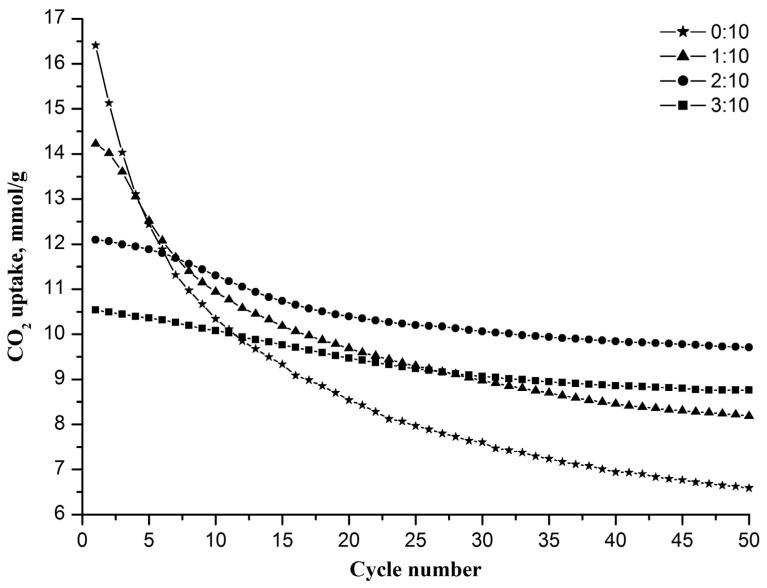
The CO_2_ uptake capacities of nanofibrous sorbents with different Zr/Ca molar ratios depending on the cycle number.

All of the fabricated nanofiber sorbents were examined in a regime of multicyclic carbonation/decarbonation (Figure 1). It was found that pure nanofibrous CaO has the highest initial CO_2_ uptake capacity of 16.4 mmol/g, which is close to the stoichiometric capacity of CaO (17.9 mmol/g). However, the capacity of pure nanofibrous CaO rapidly decreases with the increasing carbonation/decarbonation cycle number, reaching 6.6 mmol/g at the 50th cycle. Sorbents with Zr/Ca molar ratios of 1:10, 2:10, and 3:10 have lower initial capacities of 14.2, 12.1, and 10.5 mmol/g, respectively, but show higher capacity values at the 50th cycle—8.2, 9.7 and 8.8 mmol/g, respectively. The inertness of zirconia to carbon dioxide explains why the larger the Zr/Ca molar ratio, the lower the initial capacity of the nanofiber sorbent and the less it decreases during cyclic carbonation/decarbonation. The CO_2_ uptake capacity of the nanofiber sorbent reaches a steady-state value after the 40th cycle at a Zr/Ca molar ratio of 2:10 or higher.

It can be seen that the sorbent with a Zr/Ca molar ratio of 2:10 is characterized by the highest CO_2_ uptake capacity after 50 cycles among all tested sorbents. It shows the best performance in terms of capacity and cyclic stability. Therefore, in what follows, a composite sorbent refers to a sorbent with a Zr/Ca molar ratio of 2:10, hereinafter referred to as the Zr-doped CaO nanofiber sorbent.

After 50 cycles, the Zr-doped CaO nanofiber sorbent showed a competitive CO_2_ uptake capacity among Zr-doped CaO sorbents previously fabricated by various methods (Table 1).

According to Table 1, the electrospun sorbent in terms of its CO_2_ uptake capacity is inferior only to the sorbent obtained by flame spray pyrolysis. However, flame spray pyrolysis is more costly and labor-intensive than electrospinning.

**Figure 2:**
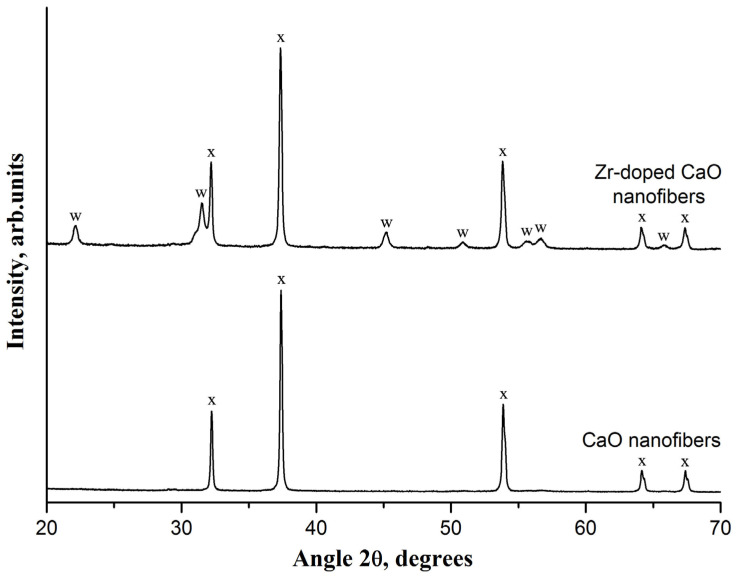
The XRD patterns of the pure CaO nanofiber sorbent and Zr-doped CaO nanofiber sorbent. The following compounds were identified: (x)—CaO; (w)—CaZrO_3_.

The XRD analysis indicates the presence of only CaO reflections at 32.2°, 37.4°, 53.9°, 64.2°, and 67.4° in the virgin CaO nanofiber sorbent (Figure 2). In addition to the CaO characteristic peaks, the XRD pattern of the virgin Zr-doped CaO nanofiber sorbent contains calcium zirconate peaks at 22.2°, 31.5°, 45.2°, 50.9°, 55.5°, 56.7°, and 65.8°, which indicates a reaction between ZrO_2_ and CaO, resulting in CaZrO_3_ formation. The absence of the most intensive characteristic ZrO_2_ peaks at 28.2°, 30.1°, 30.2°, and 31.5° in the XRD pattern of the Zr-doped CaO nanofiber sorbent indicates the complete reaction between ZrO_2_ and CaO. The formation of CaZrO_3_ is in agreement with [34], reporting the reaction of CaO and ZrO_2_ at a temperature of 800 °C. Since CaZrO_3_ is inert to CO_2_, this reaction additionally reduces the initial CO_2_ uptake capacity of the Zr-doped CaO nanofiber sorbent due to the CaO content decrease.

**Figure 3:**
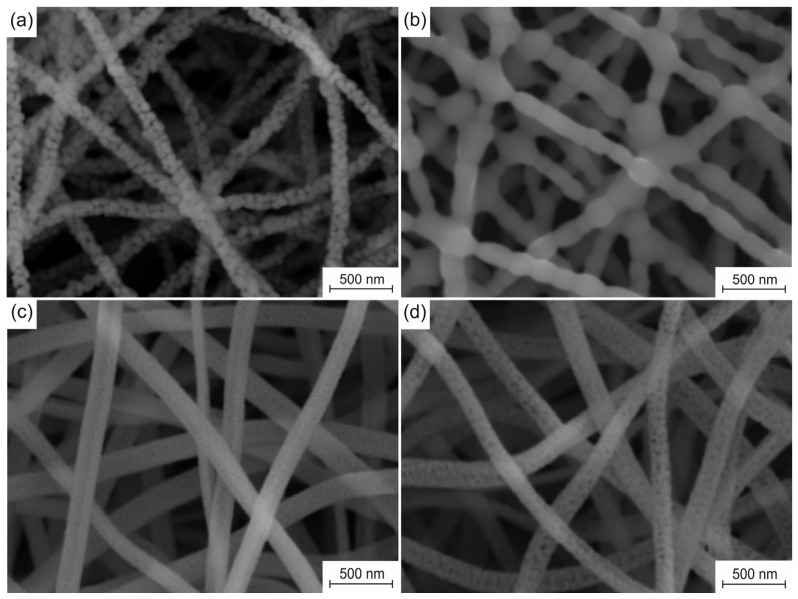
SEM images of sorbents before and after 50 cycles: (**a**,**b**) pure CaO nanofiber sorbent; (**c**,**d**) Zr-doped CaO nanofiber sorbent.

The microstructure evolution of the fabricated nanofiber sorbents caused by cyclic carbonation/decarbonation is presented in Figure 3. The fresh pure CaO nanofiber sorbent is characterized by an average filament diameter of 130 ± 11 nm, an average grain size of 74 nm, and a specific surface area of 31 m^2^/g. After the 50th carbonation/decarbonation cycle, the average diameter of the filaments increases to 151 ± 15 nm, and their specific surface area decreases to 12 m^2^/g. In addition, the obtained nanofibers have a thickness of one grain. At the same time, the virgin Zr-doped CaO nanofiber sorbent is characterized by an average filament diameter of 164 ± 16 nm, an average grain size of 15 nm, and a specific surface area of 79 m^2^/g. After the 50th carbonation/decarbonation cycle, the average diameter of its filaments increases to 158 ± 14 nm, and their specific surface area decreases to 54 m^2^/g with an increase in the average grain size to 36 nm.

The observed degradation of the microstructure of pure CaO nanofibers and a sharp decrease in their ability to chemically adsorb CO_2_ during cyclic carbonation/decarbonation can be explained by calcium carbonate sintering. The Tammann temperatures for CaO and CaCO_3_ are 1154 °C and 533 °C, respectively [35]. Thus, it can be concluded that CaCO_3_ sintering occurs not only during CaO carbonation but also when the resulting CaCO_3_ is heated to the decarbonation stage and during CaCO_3_ decarbonation. Due to the sintering of CaCO_3_, larger and larger CaO particles are formed in each subsequent calcination stage. The carbonate layer formed on the surface of the CaO particles prevents the penetration of CO_2_ deep into CaO particles, thereby locking the unreacted CaO inside the particles. Therefore, only the near-surface layer of CaO particles participates in the carbonation process, while the core is excluded from the reaction. As a result of cyclic carbonation/decarbonation, more and more CaO is removed from the carbonation reaction due to CaO particle growth (Figure 3b).

The appearance of inert and heat-resistant CaZrO_3_ in the Zr-doped CaO nanofibers due to the reaction between ZrO_2_ and CaO prevents the CaO particle growth (Figure 3d) caused by the sintering of formed CaCO_3_. The Tammann temperature of calcium zirconate is 1218 °C [35]. The CaZrO_3_ nanoparticles form a spatial barrier between neighboring CaO nanoparticles [28,29,30,31,32,33], thereby significantly reducing the degree of their contact and, therefore, preventing their sintering in the carbonation stage, upon heating to the decarbonation stage, and in the carbonation stage. The nanosized CaO grain growth inhibition prevents the release of CaO from the carbonation reaction during multicyclic carbonation-calcination. Calcium zirconate dispersed in CaO nanofibers ensures both the stability of the microstructure and the sorption capacity of the Zr-doped CaO nanofiber sorbent during cyclic carbonation/decarbonation. It should be noted that the regular macroporous structure of the electrospun sorbent improves its activity, ensuring efficient gas transport (Figure 3).

**Figure 4:**
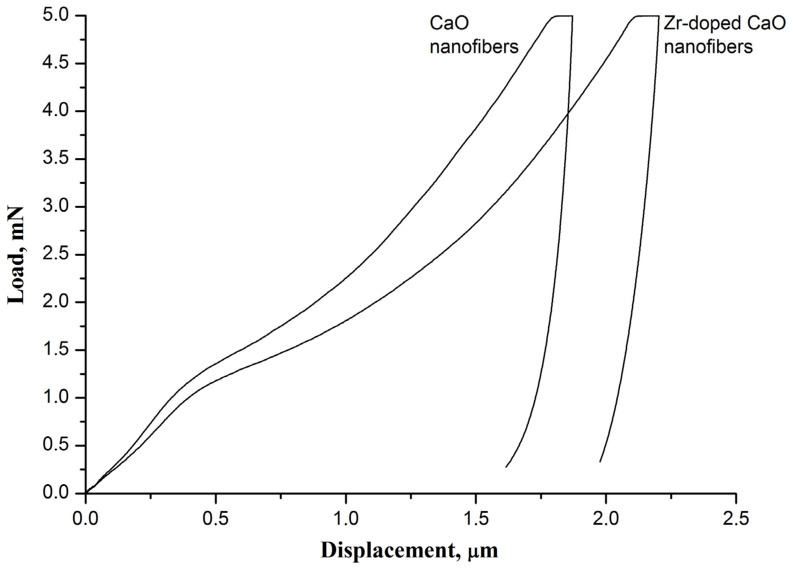
The load–displacement curves of the virgin pure CaO nanofiber sorbent and the virgin Zr-doped CaO nanofiber sorbent.

It is found that the introduction of zirconia into CaO nanofibers results in a decrease in the hardness and Young’s modulus of the nanofiber mat, which leads to an increase in its flexibility (Figure 4). If the hardness and Young’s modulus of the virgin pure CaO nanofiber sorbent fabricated at 800 °C are 1.87 ± 0.05 MPa and 786 ± 91 MPa, respectively, then the virgin Zr-doped CaO nanofiber sorbent obtained at the same temperature has a hardness of 1.60 ± 0.07 MPa and a Young’s modulus of 520 ± 25 MPa. The observed difference in the mechanical characteristics of nanofiber mats may be due to the smaller grain size in the Zr-doped CaO nanofibers compared to pure CaO nanofibers.

Since all nanofiber mats are prepared at a temperature of 800 °C, which is below the Tammann temperature of CaO, there is no sintering of the filaments at their cross-points or CaO particles inside the nanofibers. In addition, the obtained nanofibers completely lack decomposition products of the binder polymer and the precursor [25]. Therefore, the bond between CaO grains inside nanofibers is due only to Van der Waals forces, which provide the free movement of CaO grains inside them. The Zr-doped CaO nanofibers are characterized by a smaller grain size than pure CaO nanofibers (Figure 3a,c), which makes grains in the Zr-doped CaO nanofibers more mobile. In addition, the higher surface roughness of pure CaO nanofibers compared to Zr-doped CaO nanofibers due to their larger grain size negatively affects the possibility of the free movement of the filaments relative to each other. As a result, the Zr-doped CaO nanofibers and their mats are characterized by higher flexibility (and lower Young’s modulus [36]) than pure CaO nanofibers and their mats fabricated at the same temperature. The stiffer CaO nanofiber mat is more brittle and therefore has a higher hardness than the Zr-doped CaO nanofiber mat. The hardness and Young’s modulus of zirconia nanofiber mats obtained at 700–900 °C are about 1 MPa and about 150 MPa, respectively [37]. This indicates that zirconia nanofiber mats are more flexible than mats of CaO nanofibers and Zr-doped CaO nanofibers.

It is found that the pure CaO nanofiber sorbent after 50 carbonation/decarbonation cycles is characterized by a higher hardness and Young’s modulus than the virgin pure CaO nanofiber sorbent: 2.66 ± 0.12 MPa and 992 ± 83 MPa versus 1.87 ± 0.05 MPa and 786 ± 91 MPa, respectively. Figure 3b shows that cyclic carbonation/decarbonation leads to the sintering of CaO grains in pure CaO nanofibers and at the cross-points of nanofibers, which negatively affects the possibility of the free movement of the filaments. As a result, the spent pure CaO nanofiber sorbent is stiffer and more brittle than the virgin pure CaO nanofiber sorbent.

In addition, the performance of the Zr-doped CaO nanofiber sorbent in cyclic carbonation/decarbonation under various operating conditions was investigated.

**Figure 5:**
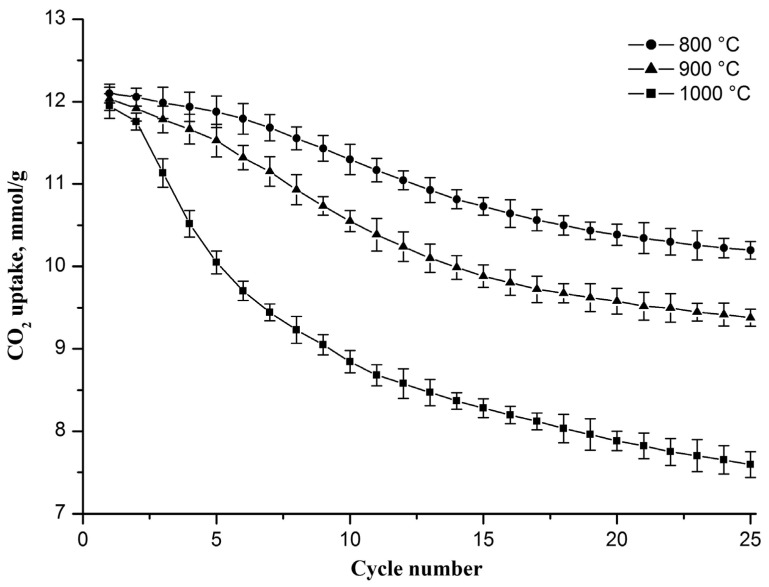
CO_2_ uptake capacity evolution of the Zr-doped CaO nanofiber sorbent during cyclic carbonation/decarbonation at different decarbonation temperatures. Each point on the graph represents the average of three measurements.

As mentioned above, CaCO_3_ sintering occurs not only during the carbonation stage but also when the resulting CaCO_3_ is heated to the decarbonation stage and during the decarbonation stage, where sintering occurs more intensively at a higher temperature, which is confirmed by the results presented in Figure 5. It can be seen that the decrease in the CO_2_ uptake capacity is more notable with each increasing cycle number at a higher decarbonation temperature. In the 25th cycle, the CO_2_ uptake capacity reaches 10.2, 9.4, and 7.6 mmol/g if decarbonation occurs at 800, 900, and 1000 °C, respectively. The drop in the capacity value increases from 1.9 to 4.3 mmol/g as the decarbonation temperature increases from 800 to 1000 °C. An increase in the decarbonation temperature to 800, 900, and 1000 °C also leads to a decrease in the specific surface area of the sorbent after 25 carbonation/decarbonation cycles, and the specific surface area of the sorbent decreases to 67, 61, and 50 m^2^/g, respectively, which indicates that the rise in the decarbonation temperature stimulates the growth of CaO grains.

Previously, a similar CO_2_ uptake capacity reduction during cyclic carbonation/decarbonation with a rise in the decarbonation temperature was observed for CaO powder sorbents functionalized with CaZrO_3_, Ca_3_Al_2_O_6_, and Nd_2_O_3_ [31,38,39].

**Figure 6:**
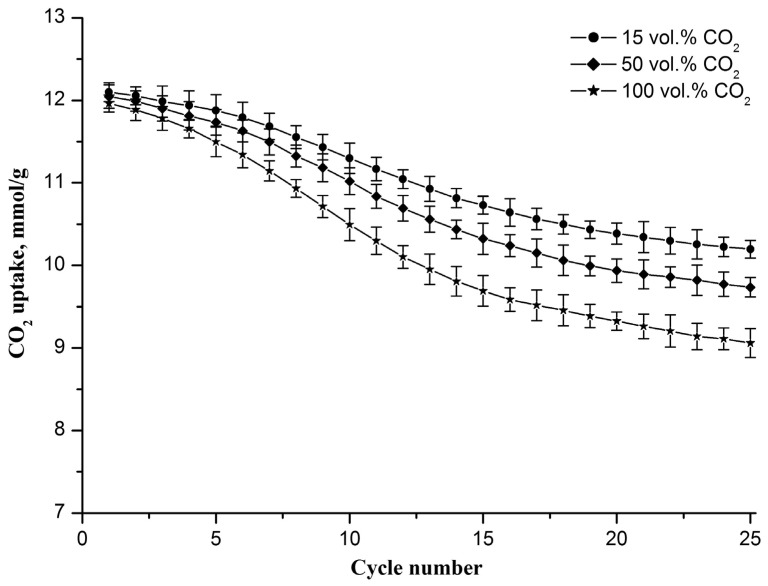
CO_2_ uptake capacity evolution of the Zr-doped CaO nanofiber sorbent during cyclic carbonation/decarbonation with different CO_2_ contents in the gas flow upon carbonation. Each point on the graph represents the average of three measurements.

The CO_2_ uptake capacity evolution of the Zr-doped CaO nanofiber sorbent during cyclic carbonation/decarbonation with different CO_2_ contents in the gas flow during the carbonation stage is shown in Figure 6. The decarbonation temperature is 800 °C. It is observed that the sorbent CO_2_ uptake capacity decreases with the increasing cycle number, reaching 10.2, 9.7, and 9.0 mmol/g in the 25th cycle at 15, 50, and 100 vol.% CO_2_, respectively. The drop in the capacity value increases from 1.9 to 3 mmol/g as the CO_2_ concentration increases from 15 to 100 vol.%.

An increase in the CO_2_ concentration in the gas flow leads to the intensification of the carbonation reaction, which results in the formation of larger CaO particles during the decomposition of CaCO_3_. This is confirmed by the specific surface area measurements. After 25 carbonation/decarbonation cycles, the specific surface area of the sorbent decreases to 67, 63, and 59 m^2^/g, respectively, as the CO_2_ concentration increases from 15 to 50 to 100 vol.%. This indicates that the Zr-doped CaO nanofiber sorbent is more resistant to cyclic carbonation/decarbonation if the carbonation occurs at lower CO_2_ concentrations.

According to results presented in Figure 5 and Figure 6, it can be seen that the sorbent CO_2_ uptake capacity tends toward smaller values both with an increase in the decarbonation temperature and with a rise in CO_2_ concentration in the gas flow during the carbonation stage. However, the sorbent CO_2_ uptake capacity is more sensitive to the decarbonation temperature than to the CO_2_ concentration in the gaseous stream during carbonation.

## 4. Conclusions

A Zr-doped CaO sorbent for high-temperature CO_2_ capture was fabricated using electrospinning. The nanofiber sorbent with an average filament diameter of about 160 nm is characterized by an initial CO_2_ uptake capacity of 12.1 mmol/g, a specific surface area of 79 m^2^/g, an indentation Young’s modulus of 520 MPa, and a hardness of 1.6 MPa. After 50 carbonation/decarbonation cycles, the sorbent has a decent CO_2_ uptake capacity of 9.7 mmol/g due to a uniform distribution of CaZrO_3_ in the CaO nanofibers to prevent CaO grain growth caused by CaCO_3_ sintering. It is revealed that the sorbent CO_2_ uptake capacity decreases both with an increase in the decarbonation temperature and with an increase in the CO_2_ concentration in the gas flow upon carbonation, where the sorbent CO_2_ uptake capacity is more sensitive to the decarbonation temperature than to the CO_2_ concentration in the gaseous stream during carbonation. It is assumed that the electrospun regenerable Zr-doped CaO sorbent is effective for removing CO_2_ from flue gases.

## Figures and Tables

**Table 1:** Sorption capacity of Zr-doped sorbents obtained by various methods.

Method	Steady-State Value of CO_2_ Uptake Capacity, mmol/g	Reference
Electrospinning	9.7	Present work
Flame spray pyrolysis	11.0	[28]
Surfactant template/ultrasound-assisted	3.4	[29]
Wet mixing	6.8	[30]
Sol–gel	7.3	[31]
Coprecipitation	5.2	[32]
Wet high-energy milling	8.6	[33]

## Data Availability

All data included in this study are available upon request from the corresponding author.
